# Design and Synthesis of 2,6-Disubstituted-4′-Selenoadenosine-5′-*N*,*N*-Dimethyluronamide Derivatives as Human A_3_ Adenosine Receptor Antagonists

**DOI:** 10.3390/ph14040363

**Published:** 2021-04-14

**Authors:** Hongseok Choi, Kenneth A. Jacobson, Jinha Yu, Lak Shin Jeong

**Affiliations:** 1 Research Institute of Pharmaceutical Sciences, College of Pharmacy, Seoul National University, Seoul 08826, Korea; choihs906@snu.ac.kr; 2 Molecular Recognition Section, Laboratory of Bioorganic Chemistry, National Institute of Diabetes and Digestive and Kidney Disease, National Institutes of Health, Bethesda, MD 20892, USA; kennethj@niddk.nih.gov; 3 Chemical Kinomics Research Center, Korea Institute of Science and Technology (KIST), Seoul 02792, Korea

**Keywords:** A_3_ adenosine receptor, structure-activity relationship, 4′-Selenonucleosides, antagonist

## Abstract

A new series of 4′-selenoadenosine-5′-*N,N*-dimethyluronamide derivatives as highly potent and selective human A_3_ adenosine receptor (hA_3_AR) antagonists, is described. The highly selective A_3_AR agonists, 4′-selenoadenosine-5′-*N*-methyluronamides were successfully converted into selective antagonists by adding a second *N*-methyl group to the 5′-uronamide position. All the synthesized compounds showed medium to high binding affinity at the hA_3_AR. Among the synthesized compounds, 2-H-*N*^6^-3-iodobenzylamine derivative **9f** exhibited the highest binding affinity at hA_3_AR. (*K_i_* = 22.7 nM). The 2-H analogues generally showed better binding affinity than the 2-Cl analogues. The cAMP functional assay with 2-Cl-*N*^6^-3-iodobenzylamine derivative **9l** demonstrated hA_3_AR antagonist activity. A molecular modelling study suggests an important role of the hydrogen of 5′-uronamide as an essential hydrogen bonding donor for hA_3_AR activation.

## 1. Introduction

Adenosine, which is the endogenous ligand of the adenosine receptors (ARs), is an important neuromodulator and mediates through activation of its four receptors, consisting of A_1_, A_2A_, A_2B_, and A_3_ subtypes. These receptors are widely distributed in tissues and involved in various physiological activities [1]. Each subtype couples to a preferred type of G protein; A_1_ and A_3_ARs primarily couple to the G_i/o_ proteins, and A_2A_ and A_2B_ARs couple to G_s_ proteins. A_2B_ and A_3_ARs are also known to be coupled to G_q_ proteins. AR signaling and their physiological roles have been extensively studied [2,3]. Among them, the A_3_AR is an important receptor to regulate cardioprotection in cardiac ischemia [4], degranulation of neutrophils [5], and cell proliferation [6]. These results led to the development of A_3_AR agonists as anticancer agents [7]. Selective A_3_AR antagonists are also promising ligands to modulate inflammation [8] and cerebroprotection [9,10]. Some studies showed that A_3_AR antagonists could enhance cancer treatment via the inhibition of HIF-1α and VEGF protein accumulation in hypoxia and in tumors [11] and are potential anti-glaucoma therapeutics as they reduce intraocular pressure in mouse and monkey [12].

**Figure 1:**
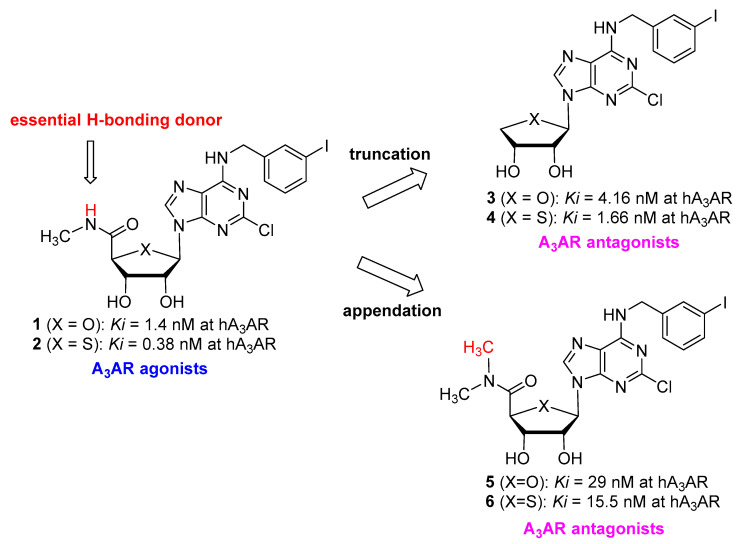
Conversion of A_3_AR agonists to A_3_AR antagonists.

In the past decades, a variety of approaches have been followed to discover novel drug candidates targeting A_3_AR. 2-Chloro-*N*^6^-(3-iodobenzyl)adenosine-5′-*N*-methyluronamide (Cl-IB-MECA, **1**) and its 4′-thio analogue **2** were discovered as potent and selective A_3_AR agonists from the extensive structure-activity relationships based on the structure of adenosine [13] (Figure 1). The 5′-uronamide hydrogen of **1**, required for full agonism, forms a putative hydrogen bond with T94 (3.36) at hA_3_AR as modeled, suggesting that this interaction is essential for receptor activation by adenosine agonists [14]. Consistent with these findings, the 4′-truncated analogues, such as **3** and **4**, lacking this hydrogen bond donor were discovered to act as A_3_AR antagonists or low-efficacy agonists, demonstrating that a hydrogen bond donating ability of the 5′-uronamide promotes A_3_AR activation [15,16]. The removal of the hydrogen bond donor ability by appending another methyl group to the 5′-uronamide, e.g., 5′-*N,N*-dimethyluronamide derivatives **5** and **6,** similarly reduced A_3_AR efficacy. These compounds were characterized as potent and selective A_3_AR antagonists [17,18].

**Figure 2:**
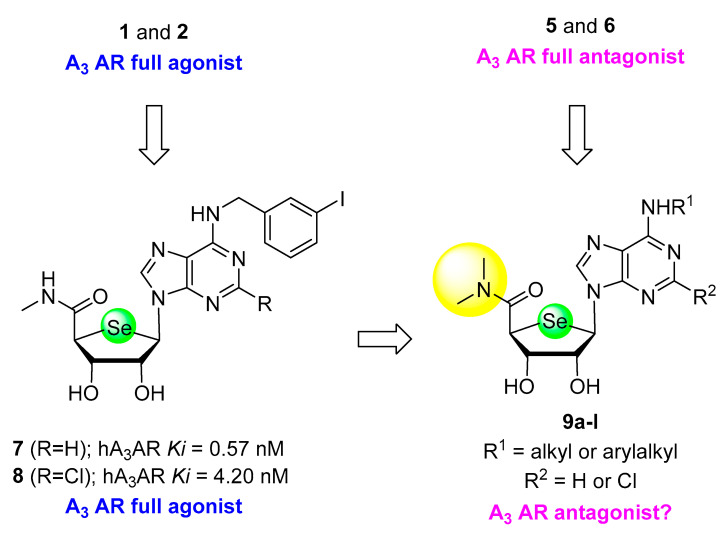
The rationale for the design of the target nucleosides **9a–l**.

On the basis of a bioisosteric rationale, we recently reported that 4′-seleno analogues of **1** and **2**, i.e., **7** and **8**, were discovered as potent and selective hA_3_AR agonists [19] (Figure 2). They exhibited comparable A_3_AR binding affinity as the corresponding 4′-oxo- and 5′-thio nucleosides **1** and **2**. However, X-ray analysis indicated that in the pure crystalline state they preferred a *syn* nucleobase orientation and a South sugar conformation, unlike **1** and **2**. As mentioned above, removal of the amide hydrogen of the 5′-uronamide of **1** and **2** by *N*-methylation, resulting in **5** and **6** successfully converted A_3_AR agonists into A_3_AR antagonists. Based on these findings, we hypothesized that the 4′-seleno analogue of **5** or **6**, bearing a 5′-*N,N*-dimethyluronamide moiety might be an A_3_AR antagonist (Figure 2). Thus, we analyzed the structure-activity relationship as A_3_AR ligands of this series by modifying *N*^6^- and C2 positions, by synthesizing novel 4′-selenonucleosides **9a–l**. Herein, we report the synthesis and biological evaluation of 2,6-disubstituted-4′-selenoadenosine-5′-*N,N*-dimethyluronamide derivatives **9a–l** as potent and selective A_3_AR a3ntagonists.

## 2. Results

### 2.1. Chemistry

**Scheme 1:**
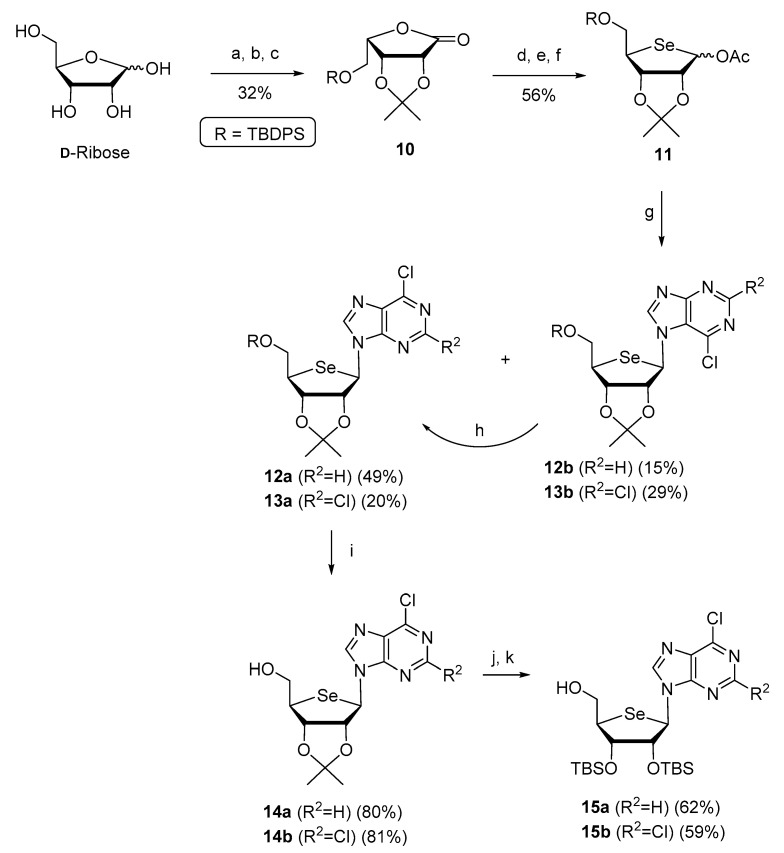
Synthesis of intermediates **15a** and **15b** from D-ribose (a) i. Br_2_, H_2_O, K_2_CO_3_; ii. Acetone, H_2_SO_4_; (b) i. MsCl, Et_3_N, CH_2_Cl_2_, 0 °C, 2 h; ii. KOH, H_2_O, rt, 15 h; (c) TBDPSCl, Et_3_N, DMAP, CH_2_Cl_2_, rt, 4 h; (d) NaBH_4_, MeOH, rt, 1 h; (e) i. MsCl, Et_3_N, CH_2_Cl_2_, rt, 1 h; ii. Se, NaBH_4_, EtOH, THF, 60 °C, 15 h; (f) i. *m*CPBA, CH_2_Cl_2_, −78 °C, 1 h; ii. Ac_2_O, 100 °C, 4 h; (g) 6-chloropurine, BSA, TMSOTf, PhCH_3_, 95 °C, 15 h or 2,6-dichloropurine, BSA, TMSOTf, CH_3_CN, 60 °C, 2 h; (h) TMSOTf, PhCH_3_, 95 °C, 30 min; (i) TBAF, THF, rt, 1 h; (j) i. PNBCl, Et_3_N, CH_2_Cl_2_, rt, 1 h; ii. TFA/H_2_O, THF, rt, 2 h; (k) i. TBSOTf, Et_3_N, DMAP, CH_2_Cl_2_, rt, 1 h; ii. NaOH, 1,4-dioxane, rt, 15 h.

For the synthesis of final compounds **9a–l**, key intermediates, 4′-seleno purine nucleosides **15a–b** were synthesized from D-ribose following the previously reported procedures [18,19] (Scheme 1). Briefly, D-ribose was converted to L-lyxonolactone derivative **10** in three steps (oxidation to lactone, conversion of D-ribo configuration to L-lyxo configuration, and *tert*-butyldiphenylsililyl (TBDPS) protection). Reduction of **10** with NaBH_4_, ring cyclization of resulting diol with selenide ion, and a Pummerer rearrangement of 4-seleno sugar afforded the glycosyl donor **11**. A Vorbrüggen condensation of **11** with 6-chloropurine and 2,6-dichloropurine produced the *N*^9^-β-anomers **12a** and **13a** with concomitant formation of their corresponding *N*^7^-β-anomers **12b** and **13b**, respectively. Conversion of *N*^7^ isomers **12b** and **13b** to their corresponding *N*^9^ isomers **12a** and **13a** was achieved by using TMSOTf at high temperature. Removal of the TBDPS group of **12a** and **13a** yielded the 5′-CH_2_OH derivatives **14a** and **14b**, respectively. Conversion of the 5′-hydroxymethyl group of **14a** and **14b** into a 5′-*N*,*N*-dimethyluronamide was successfully achieved, but the final deprotection of the acetonide group under strongly acidic conditions resulted in decomposition, instead of giving the desired final products. Thus, we exchanged the acetonide protecting group of **14a** and **14b** with a TBS group in four steps, giving **15a** and **15b**, respectively. Firstly, a PNB protecting group was attached to the 5′-position of **14a** and **14b**, followed by acetonide group deprotection with 50% aqueous TFA to give diols. The diols were then protected with a TBS group using TBSOTf followed by deprotection of the PNB group with sodium hydroxide in 1,4-dioxane to give **15a** and **15b**, respectively. The final deprotection with sodium hydroxide required mild reaction conditions (room temperature, overnight) because of the possible hydrolytic conversion of 6-chloropurine to hypoxanthine. 

**Scheme 2:**
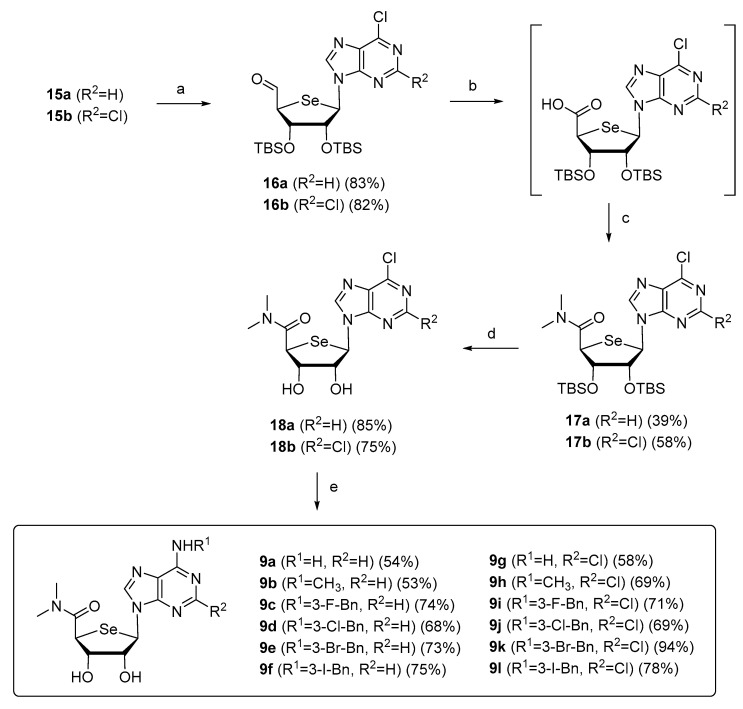
Synthesis of 5′-*N*,*N*-dimethyluronamide 4′-selenonucleoside analogues **9a–l**. (a) DMSO, Ac_2_O, 100 °C, 1 h; (b) AgNO_3_, NaOH, NH_4_OH, THF, 0 °C, 30 min; (c) (CH_3_)_2_NH∙HCl, DIPEA, HATU, THF, rt, 1 h; (d) TBAF, AcOH, THF, rt, 15 h; (e) RNH_2_, Et_3_N, EtOH, reflux, 15–30 h.

Synthesis of the final nucleosides, **9a**–**l** from the key intermediates, **15a** and **15b** is shown in Scheme 2. The direct oxidation of the alcohols of **15a** and **15b** to the carboxylic acids have been tried with many oxidizing reagents, but none of them could give the desired acid. Thus, we employed a sequential oxidation method via aldehyde instead of direct oxidation to the carboxylic acid. Albright–Goldman oxidations of **15a** and **15b**, using DMSO as an oxidizing agent under mild condition afforded the aldehydes **16a** and **16b**, respectively. Tollens′ oxidation converted the aldehydes **16a** and **16b** to the corresponding carboxylic acids smoothly, which without purification underwent the amide coupling reaction with dimethylamine in the presence of DIPEA, and HATU to yield the 5′-*N*,*N*-dimethyluronamides **17a** and **17b**, respectively. The TBS deprotection of **17a** and **17b** with TBAF and acetic acid gave the diols **18a** and **18b**, respectively. The key intermediates **18a** and **18b** were treated with various amines such as ammonia, alkylamines and 3-halobenzylamines to yield 2-H derivatives **9a**–**f** and 2-Cl derivatives **9g**–**l**, respectively.

### 2.2. Biology

#### 2.2.1. Binding Affinity

The binding affinities of all the final compounds **9a–l** were evaluated, using radioligand binding assays at four human AR subtypes (Table 1), by reported methods [20]. All of the final compounds **9a**–**l** exhibited medium to high binding affinity at the hA_3_AR, while no binding affinity at other subtypes such as hA_1_, hA_2A_, and hA_2B_ARs was observed. Among the tested compounds, compound **9f** exhibited the highest affinity (*K_i_* = 22.7 nM) at hA_3_AR, which is comparable to the corresponding 4′-oxo- and 4′-thio analogues **5** (*K_i_* = 29.0 nM) and **6** (*K_i_* = 15.5 nM). The introduction of a 3-halobenzyl group at the *N*^6^ position increased the hA_3_AR binding affinity when compared to the *N*^6^-unsubstituted adenine compounds **9a** or **9g**, indicating that a favorable hydrophobic interaction exits at the hA_3_AR binding site. In the 2-H series, the binding affinity of 3-halobenzyl derivatives **9c–f** was decreased in the following order: 3-I-benzyl **9f** > 3-Br-benzyl **9e** > 3-Cl-benzyl **9d** > 3-F-benzyl **9c**. The halogen size correlated with hA_3_AR binding affinity, whereas in the 2-Cl series, the binding affinity of 3-halobenzyl derivatives **9i**–**l** was almost same within the range of 180–250 nM. In general, the 4′-seleno-5′-*N*,*N*-dimethyluronamide derivatives **9a-l** exhibited lower binding affinity than the 4′-seleno-5′-*N*-methyluronamide derivatives **7** and **8**. It is interesting to note that 2-Cl-*N*^6^-3-iodobenzyl analogue **9l** exhibited much weaker binding affinity than the corresponding 2-H analogue **9f**. This tendency was also found in the 5′-*N*-methyluronamide 4′-seleno derivatives **7** and **8**.

#### 2.2.2. CAMP Functional Assay

**Figure 3:**
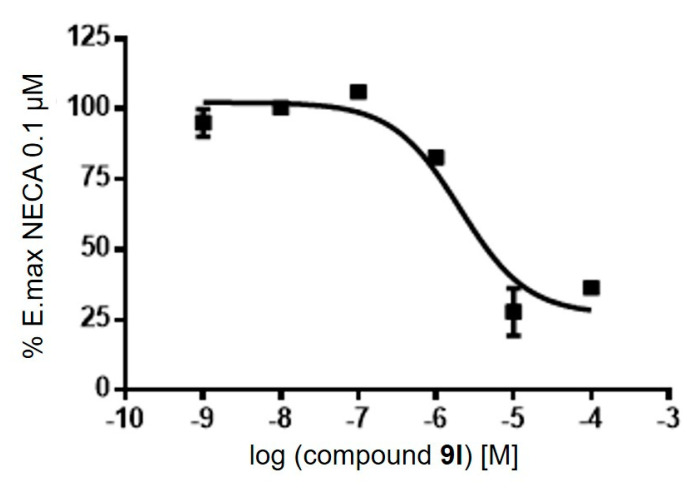
Concentration−response curve of **9l** in a functional assay at human A_3_AR measuring inhibition of 10 μM NECA-induced cAMP accumulation. Points represent mean ± SD (vertical bars) of duplicate experiments.

In a cAMP functional assay at hA_3_AR expressed in CHO cells, compound **9l** behaved as an antagonist, like compounds **5** and **6**, with K_B_ value of 114.5 nM (Figure 3). Like **5** and **6**, an additional methyl group on the 5′-*N*-methyluronamide converted an agonist into an antagonist, indicating that amide hydrogen is essential for receptor activation in this series, as well. However, the fact that the 5′-*N,N*-dimethyluronamide derivatives exhibited weaker binding affinity than the corresponding 5′-*N*-methyluronamide derivatives demonstrates that steric effects induced by 5′-*N,N*-dimethyluronamide reduce the binding affinity at the A_3_AR. 

### 2.3. Molecular Modelling Studies

**Figure 4:**
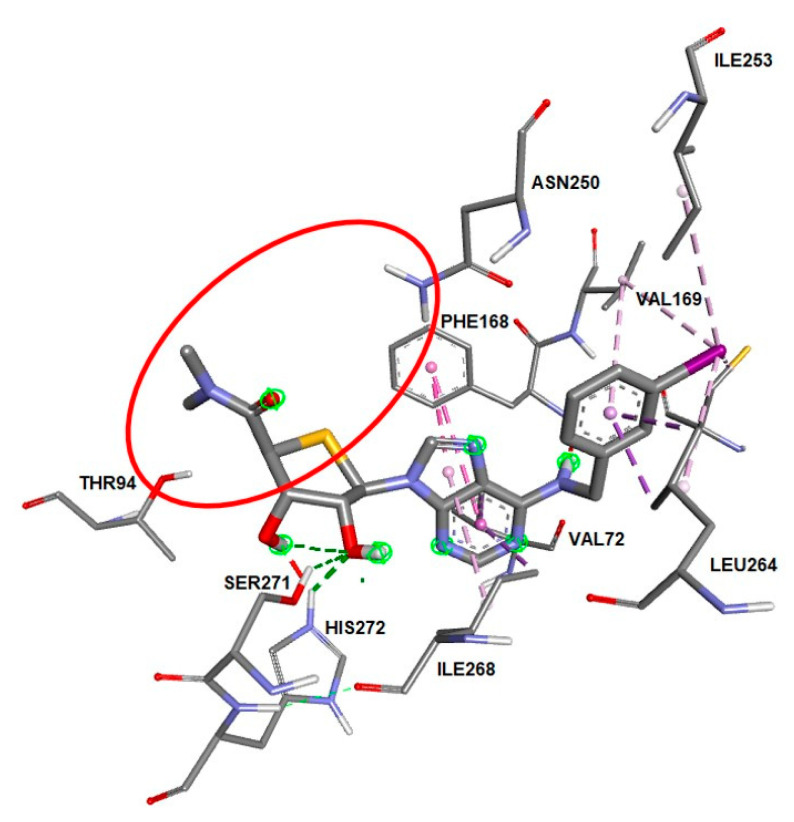
Predicted binding mode of compound **9f** in the homology model of hA_3_AR. Hydrogen bonds are depicted as green dashed line. Hydrophobic interactions are marked with a purple dashed line and π-π interactions are marked with a pink dashed line.

To investigate how 5′-*N,N*-dimethyluronamide 4′-selenonucleoside derivatives bind at hA_3_AR, we docked our compounds into the reported homology model of hA_3_AR [21] using Autodock Vina [22]. The most potent compound **9f** bound well at the orthosteric binding site with a South ring conformation (2′-endo/3′-exo), displaying H-bonds with Ser271 and His272 (Figure 4). Compared to the 5′-*N*-methyluronamide derivative **5**, the adenine ring still maintained π−π interaction with Phe168 and the iodobenzene ring had interactions with Val169, Ile253 and Leu264 [19]. The glycosidic bond was in an *anti* conformation. However, either H-bonding of 5′-*N*-uronamide with Thr94 or the adenine with Asn250 was not observed (marked as a red circle in Figure 4), suggesting that this H-bonding plays a key role in discriminating an agonist from an antagonist.

## 3. Materials and Methods

### 3.1. Chemical Synthesis

Proton (^1^H) and carbon (^13^C) NMR spectra were obtained on a Jeol JNM-ECA 300 (JEOL Ltd. Tokyo, Japan; 300/75 MHz), Bruker AV 400 (Bruker, Billerica, MA, USA; 400/100 MHz), and AMX 500 (Bruker, Billerica, MA, USA; 500/125 MHz) spectrometer. The ^1^H NMR data were reported as peak multiplicities: s for singlet; d for doublet; dd for doublet of doublets; t for triplet; td for triplet of doublet; q for quartet; quin for quintet; bs for broad singlet and m for multiplet. Coupling constants were reported in hertz. The chemical shifts were reported as ppm (δ) relative to the solvent peak. All reactions were routinely carried out under an inert atmosphere of dry nitrogen. IKA RCT basic type heating mantle was used to provide a constant heat source. Microwave-assisted reactions were carried out in sealed vessels using a Biotage Initiator + US/JPN (Biotage, Uppsala, Sweden; part no. 356007) microwave reactor, and the reaction temperatures were monitored by an external surface IR sensor. High-resolution mass spectra were measured with electrospray-ionization quadrupole time-of-flight (ESI-Q-TOF) techniques. Melting points were recorded on a Barnstead electrothermal 9100 instrument and are uncorrected. Reactions were checked by thin layer chromatography (Kieselgel 60 F254, Merck, Kenilworth, NJ, US). Spots were detected by viewing under a UV light, and by colorizing with charring after dipping in a *p*-anisaldehyde solution. The crude compounds were purified by column chromatography on a silica gel (Kieselgel 60, 70−230 mesh, Merck). All the anhydrous solvents were redistilled over CaH_2_, or P_2_O_5_, or sodium/benzophenone prior to the reaction.

(2*S*,3*S*,4*R*,5*R*)-3,4-Bis((tert-butyldimethylsilyl)oxy)-5-(6-chloro-9*H*-purin-9-yl)tetrahydroselenophene-2-carbaldehyde (**16a**). To a solution of **15a [19]** (348 mg, 0.60 mmol) in dimethyl sulfoxide (5 mL) was added acetic anhydride (0.11 mL, 1.2 mmol), and the reaction mixture was stirred at 100 °C for 1 h, cooled to room temperature, and diluted with dichloromethane (20 mL). The mixture was washed with water (10 mL × 2), and the aqueous layer was further extracted with dichloromethane (20 mL × 2). The combined organic layers were washed with brine (5 mL), dried (MgSO_4_), filtered, and concentrated under reduced pressure. The residue was purified by silica gel column chromatography (hexane–ethyl acetate = 4:1) to give **16a** (288 mg, 83%) as a white foam: ^1^H NMR (400 MHz, CDCl_3_) *δ* 9.76 (d, 1 H, *J =* 2.4 Hz), 8.78 (s, 1 H), 8.32 (s, 1 H), 6.24 (d, 1 H, *J =* 7.2 Hz), 4.86 (dd, 1 H, *J =* 2.6, 7.0 Hz), 4.68 (t, 1 H, *J =* 3.0 Hz), 4.12 (dd, 1 H, *J =* 2.4, 2.8 Hz), 0.95 (s, 9 H), 0.71 (s, 9 H), 0.12 (s, 6 H), −0.05 (s, 3 H), −0.4 (s, 3 H); ^13^C NMR (100 MHz, CDCl_3_) *δ* 194.6, 152.3, 152.1, 151.7, 145.3, 132.5, 81.0, 77.4, 74.6, 56.9, 56.8, 53.0, 25.9, 25.7, 18.3, 17.9, 0.21, −4.2, −4.45, −4.55, −5.3.

(2*S*,3*S*,4*R*,5*R*)-3,4-Bis((tert-butyldimethylsilyl)oxy)-5-(2,6-dichloro-9*H*-purin-9-yl)tetrahydroselenophene-2-carbaldehyde (**16b**). Compound **15b [19]** (1.49 g, 2.43 mmol) was converted to **16b** (1.22 g, 82%) as a yellow foam, using a procedure similar to that used in the preparation of **16a**: ^1^H NMR (500 MHz, CDCl_3_) *δ* 9.73 (d, *J =* 2.1 Hz, 1 H), 8.40 (s, 1 H), 6.13 (d, *J =* 6.8 Hz, 1 H), 4.74−4.73 (m, 1 H), 4.63 (t, *J =* 2.9 Hz, 1 H), 4.11 (s, 1 H), 0.91 (s, 9 H), 0.71 (s, 9 H), 0.090 (s, 6 H), −0.043 (s, 3 H), −0.38 (s, 3 H); ^13^C NMR (75 MHz, CDCl_3_) *δ* 194.3, 153.2, 153.0, 152.2, 145.7, 131.3, 81.0, 74.4, 56.7, 52.9, 25.69, 25.66, 25.6, 18.0, 17.6, −4.42, −4.70, −4.81, −4.86, −5.55.

(2*S*,3*S*,4*R*,5*R*)-3,4-Bis((tert-butyldimethylsilyl)oxy)-5-(6-chloro-9*H*-purin-9-yl)-*N*-methyltetrahydroselenophene-2-carboxamide (**17a**). To a solution of AgNO_3_ (170 mg, 1.00 mmol) was added 1 N NaOH (1.0 mL, 1.00 mmol) at 0 °C. After Ag_2_O was precipitated, 24% ammonia−water (0.3 mL) was added to the reaction mixture until the mixture was clear. The solution of **16a** (288 mg, 0.50 mmol) in THF (8 mL) was added to the above Tollens’ reagent at 0 °C, and the reaction mixture was stirred at the same temperature for 30 min and diluted with water (10 mL). The aqueous layer was extracted with ethyl acetate (10 mL × 2), and the aqueous layer was acidified with 1 N HCl solution (3 mL) and extracted with ethyl acetate (10 mL × 2). The combined organic layers were washed with brine, dried (MgSO_4_), filtered, and evaporated under reduced pressure to give the crude acid. To a solution of the crude acid in THF (5 mL) were added 1-[bis(dimethylamino)methylene]-1*H*-1,2,3-triazolo[4,5-*b*]-pyridinium 3-oxide hexafluorophosphate (HATU) (190 mg, 0.50 mmol), dimethylamine hydrochloride (82 mg, 1.00 mmol), and diisopropylamine (0.19 mL, 1.10 mmol) at room temperature. The reaction mixture was stirred at same temperature for 1 h and diluted with ethyl acetate (10 mL). The organic layer was washed with water (5 mL × 2), and the aqueous layer was further extracted with ethyl acetate (10 mL × 2). The combined organic layers were washed with brine (5 mL), dried (MgSO_4_), filtered, and concentrated under reduced pressure. The residue was purified by silica gel column chromatography (hexane–ethyl acetate = 3:1) to give **17a** (120 mg, 39%) as a white foam; ^1^H NMR (400 MHz, CDCl_3_) *δ* 8.76 (s, 1 H), 8.67 (bs, 1 H), 6.32 (d, *J =* 6.8 Hz, 1 H), 4.9 (bs, 1 H), 4.63 (m, 1 H), 4.21 (m, 1 H), 3.04 (s, 3 H), 2.99 (s, 3 H), 0.92 (s, 9 H), 0.71 (s, 9 H), 0.08 (d, J = 3.6 Hz, 6 H), 0.01 (s, 3 H)

(2*S*,3*S*,4*R*,5*R*)-3,4-Bis((tert-butyldimethylsilyl)oxy)-5-(2,6-dichloro-9*H*-purin-9-yl)-*N*-methyltetrahydroselenophene-2-carboxamide (**17b**). Compound **16b** (623 mg, 1.02 mmol) was converted to **17b** (260 mg, 58%) as a yellow foam, using a procedure similar to that used in the preparation of **17a**: ^1^H NMR (400 MHz, MeOD) *δ* 8.69 (s, 1 H), 6.20 (d, *J =* 6.4 Hz, 1 H), 4.75–4.85 (bs, 1 H), 4.60 (m, 1 H), 4.22 (m, 1 H), 3.02 (s, 3 H), 2.98 (s, 3 H), 0.90 (s, 9 H), 0.74 (s, 9 H), 0.06 (s, 6 H), 0.02 (s, 3 H)

(2*S*,3*S*,4*R*,5*R*)-5-(6-Chloro-9*H*-purin-9-yl)-3,4-dihydroxy-*N*-methyltetrahydro selenophene-2-carboxamide (**18a**). To a stirred solution of **17a** (80 mg, 0.13 mmol) in THF (3 mL) was added 1 M tetra-n-butylammonium fluoride in THF solution (0.13 mL, 0.13 mmol) and acetic acid (7 μL, 0.13 mmol), and the reaction mixture was stirred at room temperature for 15 h. The solvent was evaporated, and the resulting residue was purified by silica gel column chromatography (dichloromethane–methanol = 100:1−10:1) to give **18a** (43 mg, 85%) as a white solid; ^1^H NMR (400 MHz, CDCl_3_) *δ* 8.93 (s, 1 H), 8.73 (s, 1 H), 6.37 (d, *J =* 6.4 Hz, 1 H), 4.95 (dd, *J =* 6 Hz, 3.2 Hz, 1 H), 4.67 (t, *J =* 3.8 Hz, 1 H), 4.50 (d, *J =* 4 Hz, 1 H), 3.03 (s, 3 H), 2.99 (s, 3 H).

(2*S*,3*S*,4*R*,5*R*)-5-(2,6-Dichloro-9*H*-purin-9-yl)-3,4-dihydroxy-*N*-methyltetra hydroselenophene-2-carboxamide (**18b**). Compound **17b** (83 mg, 0.127 mmol) was converted to **18b** (41 mg, 75%) as a white solid, using a procedure similar to that used in the preparation of **18a**; ^1^H NMR (400 MHz, MeOD) *δ* 8.93 (s, 1 H), 6.27 (d, *J =* 6 Hz, 1 H), 4.88 (m, 1 H), 4.65 (t, *J =* 4 Hz, 1 H), 4.50 (d, *J =* 4.4 Hz, 1 H), 3.04 (s, 3 H), 2.99 (s, 3 H).

#### General Procedure for the Synthesis of **9a**–**l**

To a stirred solution of **18a−b** (1 equiv.) in ethanol were added amine (3 equiv.) and triethylamine (6 equiv.), and the reaction mixture was stirred at 95 °C for 15−30 h. All volatiles were evaporated, and the residue was purified by silica gel column chromatography (dichloromethane–methanol = 100:1−10:1) to give **9a-l** as a white solid.

(2*S*,3*S*,4*R*,5*R*)-5-(6-amino-9*H*-purin-9-yl)-3,4-dihydroxy-*N,N*-dimethyltetra hydroselenophene-2-carboxamide (**9a**). White solid; yield: 54%; ^1^H NMR (400 MHz, MeOD) *δ* 8.50 (s, 1 H), 8.19 (s, 1 H), 6.24 (d, *J =* 5.6 Hz, 1 H), 4.68 (t, *J =* 4 Hz. 1 H), 4.49 (d, *J =* 4.8 Hz, 3.03 (s, 3 H), 2.98 (s, 3 H); ^13^C NMR (100 MHz, MeOD) *δ* 173.9, 157.5, 154.1, 151.3, 142.0, 120.4, 82.8, 77.7, 56.8, 42.4, 38.3, 36.5; HRMS (FAB) found 373.0537 (calculated for C_12_H_17_N_6_O_3_Se (M + H)^+^ 373.0527).

(2*S*,3*S*,4*R*,5*R*)-3,4-dihydroxy-*N,N*-dimethyl-5-(6-(methylamino)-9*H*-purin-9-yl)tetrahydroselenophene-2-carboxamide (**9b**). White solid; yield: 53%; ^1^H NMR (400 MHz, MeOD) *δ* 8.43 (s, 1 H), 8.23 (s, 1 H), 6.23 (d, *J =* 6 Hz, 1 H), 4.67 (t, *J =* 4 Hz, 1 H), 4.48 (d, *J =* 4.4 Hz, 1 H), 3.02 (s, 3 H), 2.97 (s, 3 H); ^13^C NMR (100 MHz, MeOD) *δ* 173.89, 157.0, 154.1, 149.6, 141.3, 120.9, 82.0, 77.6, 62.4, 56.8, 42.4, 38.3, 36.4; HRMS (FAB) found 387.0675 (calculated for C_13_H_19_N_6_O_3_Se (M + H)^+^ 387.0684).

(2*S*,3*S*,4*R*,5*R*)-5-(6-((3-fluorobenzyl)amino)-9*H*-purin-9-yl)-3,4-dihydroxy-*N,N*-dimethyltetrahydroselenophene-2-carboxamide (**9c**). White solid; yield: 74%; ^1^H NMR (400 MHz, MeOD) *δ* 8.46 (s, 1 H) 8.23 (s, 1 H), 7.28 (q, *J =* 6 Hz, 1 H) 7.15 (d, *J =* 7.6 Hz, 1 H), 7.07 (d, *J =* 9.6 Hz, 1 H), 6.92 (td, *J =* 8, 2 Hz, 1 H), 6.24 (d, *J =* 5.6 Hz, 1 H), 4.68 (dd, *J =* 4.4, 4 Hz, 1 H) 4.48 (d, *J =* 4.8 Hz, 1 H) 3.02 (s, 3 H), 2.97 (s, 3H); ^13^C NMR (100 MHz, MeOD) *δ* 173.9, 165.8, 163.3, 156.2, 154.2, 150.7, 143.5, 141.6, 131.4, 124.3, 120.8, 115.1, 82.1, 77.6, 56.8, 42.4, 38.3, 36.4; HRMS (FAB) found 481.0894 (calculated for C_19_H_22_FN_6_O_3_Se (M + H)^+^ 481.0903).

(2*S*,3*S*,4*R*,5*R*)-5-(6-((3-chlorobenzyl)amino)-9*H*-purin-9-yl)-3,4-dihydroxy-*N,N*-dimethyltetrahydroselenophene-2-carboxamide (**9d**). White solid; yield: 68%; ^1^H NMR (400 MHz, MeOD) *δ* 8.46 (s, 1 H), 8.24 (s, 1 H), 7.36 (s, 1 H), 7.18–7.28 (m, 4 H), 6.24 (d, *J =* 5.2 Hz, 1 H), 4.68 (dd, *J =* 4.4 Hz, 4 Hz, 1 H), 4.48 (d, *J =* 4.8 Hz, 1 H), 3.02 (s, 3 H), 2.97 (s, 3 H); ^13^C NMR (100 MHz, MeOD) *δ* 173.8, 156.1, 154.2, 143.1, 141.7, 135.5, 131.2, 128.6, 128.3, 128.3, 127.0, 120.8, 82.0, 77.6, 56.9, 42.4, 38.2, 36.4; HRMS (FAB) found 497.0599 (calculated for C_19_H_22_ClN_6_O_3_Se (M + H)^+^ 497.0607).

(2*S*,3*S*,4*R*,5*R*)-5-(6-((3-bromobenzyl)amino)-9*H*-purin-9-yl)-3,4-dihydroxy-*N,N*-dimethyltetrahydroselenophene-2-carboxamide (**9e**). White solid; yield: 75%, ^1^H NMR (400 MHz, MeOD + CDCl_3_ = 1:3); *δ* 8.42 (s, 1 H), 8.29 (s, 1 H), 7.50 (s, 1 H), 7.36 (d, *J =* 7.6 Hz, 1 H), 7.28 (d, *J =* 7.6 Hz, 1 H), 7.17 (t, *J =* 8 Hz, 1 H), 6.17 (d, *J =* 4.8 Hz, 1 H), 4.76 (bs, 2 H), 4.73–4.69 (m, 1 H), 4.44 (d, 4.8 Hz, 1 H), 3.02 (s, 3 H), 2.98 (s, 3 H); ^13^C NMR (100 MHz, MeOD + CDCl_3_ = 1:3) *δ* 172.1, 155.0, 153.3, 141.2, 140.6, 130.8, 130.7, 130.5, 126.5, 122.9, 119.8, 81.3, 77.8, 76.6, 55.9, 41.5, 38.0, 36.3; HRMS (FAB) found 541.0106 (calculated for C_19_H_21_BrN_6_O_3_Se (M + H)^+^ 541.0102).

(2*S*,3*S*,4*R*,5*R*)-3,4-dihydroxy-5-(6-((3-iodobenzyl)amino)-9*H*-purin-9-yl)-*N,N*-dimethyltetrahydroselenophene-2-carboxamide (**9f**). White solid; yield: 73%, ^1^H NMR (400 MHz, MeOD + CDCl_3_ =1:3) *δ* 8.45 (s, 1 H), 8.27 (s, 1 H), 7.71 (s, 1 H), 7.56 (d, *J =* 7.4 Hz, 1 H), 7.33 (d, *J =* 7.7 Hz, 1 H), 7.04 (t, *J =* 7.8 Hz, 1 H), 6.21 (d, *J =* 5.4 Hz, 1 H), 4.78–4.76 (m, 1 H), 4.74 (bs, 2 H), 4,71–4.69 (m, 1 H), 4.46 (d, *J =* 4.9 Hz, 1 H), 3.03 (s, 3 H), 2.99 (s, 3 H); ^13^C NMR (100 MHz, DMSO-*d*^6^) *δ* 173.5, 156.1, 154.3, 142.5, 141.7, 137.8, 137.7, 131.6, 128.1, 120.9, 95.6, 82.3, 79.2, 78.8, 77.7, 57.0, 42.5, 38.9, 37.2; HRMS (FAB) found 588.9971 (calculated for C_19_H_21_IN_6_O_3_Se (M + H)^+^ 588.9963).

(2*S*,3*S*,4*R*,5*R*)-5-(2-chloro-6-amino-9*H*-purin-9-yl)-3,4-dihydroxy-*N,N*-dimethyltetrahydroselenophene-2-carboxamide (**9g**). White solid; yield: 58%; ^1^H NMR (400 MHz, DMSO-*d*^6^) *δ* 8.33 (s, 1 H), 5.98 (d, *J =* 4.8 Hz, 1 H), 5.78 (d, *J =* 4 Hz, 1 H), 5.48 (d, *J =* 3.2 Hz, 1 H), 4.74 (bs, 1 H), 4.50 (bs, 1 H), 4.32 (d, *J =* 2.8 Hz, 1 H), 2.91 (s, 3 H), 2.87 (s, 3 H); ^13^C NMR (100 MHz, DMSO-*d*^6^) *δ* 173.7, 157.5, 154.1, 151.3, 141.9, 120.3, 81.9, 77.5, 49.6, 42.2, 38.1, 36.3; HRMS (FAB) found 407.0132 (calculated for C_12_H_16_ClN_6_O_3_Se (M + H)^+^ 407.0138).

(2*S*,3*S*,4*R*,5*R*)-5-(2-chloro-6-(methylamino)-9*H*-purin-9-yl)-3,4-dihydroxy-*N,N*-dimethyltetrahydroselenophene-2-carboxamide (**9h**). White solid; yield: 69%; ^1^H NMR (400 MHz, DMSO-*d*^6^) *δ* 8.33 (s, 1 H), 5.98 (d, *J =* 4.8 Hz, 1 H), 5.78 (d, *J =* 4 Hz, 1 H), 5.48 (d, *J =* 3.2 Hz, 1 H), 4.74 (bs, 1 H), 4.50 (bs, 1 H), 4.32 (d, *J =* 2.8 Hz, 1 H), 2.91 (s, 3 H), 2.87 (s, 3 H); ^13^C NMR (100 MHz, DMSO-*d*^6^) *δ* 171.0, 155.5, 153.4, 149.8, 139.6, 118.3, 79.5, 75.4, 64.9, 55.1, 41.1, 37.1, 35.3; HRMS (FAB) found 421.0293 (calculated for C_13_H_18_ClN_6_O_3_Se (M + H)^+^ 421.0294).

(2*S*,3*S*,4*R*,5*R*)-5-(2-chloro-6-((3-fluorobenzyl)amino)-9*H*-purin-9-yl)-3,4-dihydroxy-*N,N*-dimethyltetrahydroselenophene-2-carboxamide (**9i**). White solid; yield: 71%; ^1^H NMR (400 MHz, MeOD) *δ* 8.43 (s, 1 H), 7.29 (m, 1 H), 7.17 (d, *J =* 6.8 Hz, 1 H), 7.09 (d, *J =* 10 Hz, 1 H), 6.94 (m, 1 H), 6.15 (d, *J =* 5.2 Hz, 1 H), 4.76 (m, 1 H), 4.66 (t, *J =* 4 Hz, 1 H), 4.47 (d, *J =* 5.2 Hz, 1 H); ^13^C NMR (100 MHz, MeOD) *δ* 173.8, 165.7, 163.3, 156.5, 152.0, 143.5, 142.0, 131.4, 129.7, 126.5, 124.6, 115.3, 82.1, 77.6, 57.0, 42.4, 38.3, 36.5; HRMS (FAB) found 515.0518 (calculated for C_19_H_21_ClFN_6_O_3_Se (M + H)^+^ 515.0513).

(2*S*,3*S*,4*R*,5*R*)-5-(2-chloro-6-((3-chlorobenzyl)amino)-9*H*-purin-9-yl)-3,4-dihydroxy-*N,N*-dimethyltetrahydroselenophene-2-carboxamide (**9j**). White solid; yield: 69%; ^1^H NMR (400 MHz, MeOD) *δ* 8.43 (s 1 H), 7.38 (s, 1 H), 7.21–7.30 (m, 3 H), 6.15 (d, *J =* 5.6 Hz, 1 H), 4.76 (dd, *J =* 5.6 Hz, 3.2 Hz, 1 H), 4.71 (bs, 1 H), 4.65 (dd, *J =* 4.8 Hz, 3.2 Hz, 1 H), 4.47 (d, 5.2 Hz, 1 H), 3.03 (s, 3 H), 2.98 (s, 3 H); ^13^C NMR (100 MHz, MeOD) *δ* 173.8, 156.6, 155.7, 152.0, 142.7, 142.0, 135.5, 131.2, 128.9, 128.4, 127.3, 119.3, 82.1, 77.6, 57.0, 44.7, 42.4, 38.3, 36.5; HRMS (FAB) found 531.0211 (calculated for C_19_H_21_Cl_2_N_6_O_3_Se (M + H)^+^ 531.0217).

(2*S*,3*S*,4*R*,5*R*)-5-(6-((3-bromobenzyl)amino)-2-chloro-9*H*-purin-9-yl)-3,4-dihydroxy-*N,N*-dimethyltetrahydroselenophene-2-carboxamide (**9k**). White solid; yield: 94%; ^1^H NMR (400 MHz, MeOD) *δ* 8.43 (s, 1 H), 7.54 (s, 1 H), 7.36 (m, 2 H), 7.33–7.39 (t, *J =* 7.6 Hz, 1 H), 6.15 (d, *J =* 5.6 Hz, 1 H), 4.76 (dd, *J =* 6.4 Hz, 3.2 Hz, 1 H), 4.71 (bs, 1 H), 4.65 (dd, *J =* 3.2 Hz, 1 H), 4.48 (d, *J =* 4.8 Hz, 1 H), 3.03 (s, 3 H), 2.98 (s, 3 H); ^13^C NMR (100 MHz, MeOD) *δ* 174.5, 157.3, 156.5, 152.7, 143.6, 142.7, 132.6, 132.1, 128.4, 124.2, 82.8, 78.3, 57.6, 45.3, 43.0, 38.9, 37.1; HRMS (FAB) found 574.9708 (calculated for C_19_H_21_BrClN_6_O_3_Se (M + H)^+^ 574.9712).

(2*S*,3*S*,4*R*,5*R*)-5-(2-chloro-6-((3-iodobenzyl)amino)-9*H*-purin-9-yl)-3,4-dihydroxy-*N,N*-dimethyltetrahydroselenophene-2-carboxamide (**9l**). White solid; yield: 78%; ^1^H NMR (400 MHz, MeOD) *δ* 8.43 (s, 1 H), 7.75 (s, 1 H), 7.57 (d, *J =* 4 Hz, 1 H), 7.36 (d, *J =* 3.6 Hz, 1 H), 7.07 (t, *J =* 8 Hz, 1 H), 6.15 (d, *J =* 5.6 Hz, 1 H), 4.76 (dd, *J =* 5.2 Hz, 3.2 Hz, 1 H), 4.64–4.68 (m, 2 H), 4.48 (d, *J =* 4.8 Hz, 1 H), 3.03 (s, 3 H), 2.98 (s, 3 H); ^13^C NMR (100 MHz, MeOD) *δ* 173.8, 156.8, 155.9, 152.0, 142.8, 142.0, 138.0, 137.6, 131.5, 128.3, 95.1, 82.1, 77.6, 57.0, 44.5, 42.4, 38.3, 36.5.; HRMS (FAB) found 622.9584 (calculated for C_19_H_21_ClIN_6_O_3_Se (M + H)^+^ 622.9574).

### 3.2. Biological Evaluation

#### 3.2.1. Binding Assay at hA_1_AR

Adenosine A_1_ receptor competition binding experiments were carried out in membranes from CHO-A_1_ cells (Euroscreen, Gosselies, Belgium). On the day of assay, membranes were defrosted and re-suspended in incubation buffer 20 mM Hepes, 100 mM NaCl, 10 mM MgCl_2_, 2 UI/mL adenosine deaminase (pH = 7.4). Each reaction well of a GF/C multiscreen plate (Millipore, Madrid, Spain), prepared in duplicate, contained 15 μg of protein, 2 nM [^3^H]DPCPX and test compound. Nonspecific binding was determined in the presence of 10 μM (R)-PIA. The reaction mixture was incubated at 25 °C for 60 min, after which samples were filtered and measured in a microplate beta scintillation counter (Microbeta Trilux, Perkin Elmer, Madrid, Spain).

#### 3.2.2. Binding Assay at hA_2A_AR

Adenosine A_2A_ receptor competition binding experiments were carried out in membranes from HeLa-A_2A_ cells. On the day of assay, membranes were defrosted and re-suspended in incubation buffer 50 mM Tris-HCl, 1 mM EDTA, 10 mM MgCl_2_ and 2 UI/mL adenosine deaminase (pH = 7.4). Each reaction well of a GF/C multiscreen plate (Millipore, Madrid, Spain), prepared in duplicate, contained 10 μg of protein, 3 nM [^3^H]ZM241385 and test compound. Nonspecific binding was determined in the presence of 50 μM NECA. The reaction mixture was incubated at 25 °C for 30 min, after which samples were filtered and measured in a microplate beta scintillation counter (Microbeta Trilux, Perkin Elmer, Madrid, Spain).

#### 3.2.3. Binding Assay at hA_2B_AR

Adenosine A_2B_ receptor competition binding experiments were carried out in membranes from HEK-293-A_2B_ cells (Euroscreen, Gosselies, Belgium) prepared following the provider’s protocol. On the day of assay, membranes were defrosted and re-suspended in incubation buffer 50 mM Tris-HCl, 1 mM EDTA, 10 mM MgCl_2_, 0.1 mM benzamidine, 10 μg/mL bacitracine and 2 UI/mL adenosine deaminase (pH = 6.5). Each reaction well prepared in duplicate contained 18 μg of protein, 35 nM [^3^H]DPCPX and test compound. Nonspecific binding was determined in the presence of 400 μM NECA. The reaction mixture was incubated at 25 °C for 30 min, after which samples were filtered through a multiscreen GF/C microplate and measured in a microplate beta scintillation counter (Microbeta Trilux, Perkin Elmer, Madrid, Spain).

#### 3.2.4. Binding Assay at hA_3_AR

Adenosine A_3_ receptor competition binding experiments were carried out in a multiscreen GF/B 96-well plate (Millipore, Madrid, Spain) pretreated with binding buffer (Tris-HCl 50 mM, EDTA 1 mM, MgCl_2_ 5 mM, 2 U/mL adenosine deaminase, pH = 7.4). In each well was incubated 30 μg of membranes from Hela-A_3_ cell line and prepared in laboratory (Lot: A005/05-07-2019, protein concentration = 3925 μg/mL), 10 nM [^3^H]-NECA (26.3 Ci/mmol, 1 mCi/mL, Perkin Elmer NET811250UC) and compounds studied in standard methods. Nonspecific binding was determined in the presence of R-PIA 100μM (Sigma P4532, Sigma-Aldrich, St. Louis, MO, USA). The reaction mixture (Vt: 200 μL/well) was incubated at 25 °C for 180 min, after filtered and washed six times with 250 μL wash buffer (Tris-HCl 50mM pH = 7.4), before measuring in a microplate beta scintillation counter (Microbeta Trilux, PerkinElmer, Madrid, Spain).

#### 3.2.5. Cyclic AMP Accumulation Assay

Human adenosine A_3_ receptor functional experiments were carried out in CHO-A_3_#18 cell line. The day before the assay, the cells were seeded on the 96-well culture plate (Falcon 353072, Corning, Glendale, AZ, USA). The cells are washed with wash buffer (Dulbecco’s modified eagle’s medium nutrient mixture F-12 ham (Sigma D8062), 25 mM Hepes; pH = 7.4). Wash buffer is replaced by incubation buffer (Dulbecco’s modified eagle’s medium nutrient mixture F-12 ham (Sigma D8062), 25 mM Hepes, 30 μM Rolipram (Sigma R6520); pH = 7.4). Test compounds and MRS1220 as reference compound (Sigma M228) are added and the cells incubated at 37 °C for 15 min. After, 0.1 μM of 5′-*N*-ethylcarboxamido-adenosine (NECA) (Sigma E2387) is added and the cells incubated at 37 °C for 10 min. Forskolin (Sigma F3917, Sigma-Aldrich, St. Louis, MO, USA) is added and incubated at 37 °C for 5 min. After incubation, the amount of cAMP is determined using a cAMP Biotrak Enzymeimmunoassay (EIA) System Kit (GE Healthcare RPN225, GE Healthcare, Chicago, IL, USA).

### 3.3. Molecular Modelling

The hA_3_AR homology model was obtained from reference 20. Autodock Vina 4 (The Scripps Research Institute, La Jolla, CA, USA) was used as the docking tool to generate ligand-protein complex using the following settings: center_x = −7.288, center_y = −8.071, center_z = 51.576, size_x = 40, size_y = 40, size_z = 40, energy_range = 4, exhaustiveness = 8. The ligand–protein complex with the best IFD score were selected and analyzed. The molecular graphic figures were generated by Biovia Discovery Studio Visualizer software (https://3dsbiovia.com/) (accessed on 13 April 2021.).

## 4. Conclusions

On the basis of potent and selective antagonist **5** and **6** at the human A_3_AR, *N*^6^-substituted-5′-*N,N*-dimethylcarbamoyl-4′-selenonuceloside derivatives (**9a–l**) were synthesized from D-ribose and evaluated for their binding affinity toward hARs. All final compounds exhibited medium to high binding affinity toward A_3_AR with high selectivity compared to other subtypes. Among these derivatives, compound **9f** was found to show the highest binding affinity (*K_i_* = 22.7 nM) at hA_3_AR, comparable to the corresponding 4′-oxo- and 4′-thio analogues **5** (*K_i_* = 29.0 nM) and **6** (*K_i_* = 15.5 nM). As in the case of 4′-oxo- and 4′-thio analogues **5** and **6**, addition of another methyl group to the 5′-*N*-methyluronamide converted an A_3_AR agonist into an A_3_AR antagonist, demonstrating the importance of amide hydrogen for receptor activation, which was supported by the molecular modelling study.

We believe that this study helps to define the pharmacophore needed for receptor activation or inactivation and will aid in the design of selective A_3_AR ligands by medicinal chemists.

## Figures and Tables

**Table 1:** Binding affinities of known A_3_AR ligands and 5′-*N,N*-dimethyluronamide-4′-selenonucleoside derivatives (**9a–l**) at human A_1_, A_2A_, A_2B_, and A_3_ARs.

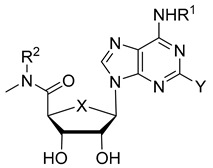								
Compound					Affinity, *K_i_*, nM ± SEM ^a,b^ (or % Inhibition at 10 uM)			
	X	Y	R^1^	R^2^	hA_1_AR	hA_2A_AR	hA_2B_AR	hA_3_AR
**5** ^c^	O	Cl	3-I-Bn	CH_3_	5870 ± 930	>10,000	>10,000	29.0 ± 4.9
**6** ^c^	S	Cl	3-I-Bn	CH_3_	6220 ± 640	>10,000	>10,000	15.5 ± 3.1
**7** ^d^	Se	H	3-I-Bn	H	480 ± 94	1080 ± 140	ND	0.57 ± 0.10
**8** ^d^	Se	Cl	3-I-Bn	H	311 ± 47	1200 ± 70	ND	4.20 ± 0.73
**9a**	Se	H	H	CH_3_	16% ± 4	22% ± 3	17% ± 1	3710 ± 600
**9b**	Se	H	CH_3_	CH_3_	9% ± 2	3% ± 2	9% ± 4	609 ± 47
**9c**	Se	H	3-F-Bn	CH_3_	13% ± 2	12% ± 1	19% ± 5	2020 ± 170
**9d**	Se	H	3-Cl-Bn	CH_3_	23% ± 5	4% ± 3	15% ± 1	1190 ± 160
**9e**	Se	H	3-Br-Bn	CH_3_	43% ± 7	37% ± 8	ND	36.3 ± 12.7
**9f**	Se	H	3-I-Bn	CH_3_	36% ± 6	32% ± 1	ND	22.7 ± 8.9
**9g**	Se	Cl	H	CH_3_	9% ± 1	3% ± 3	25% ± 5	3250 ± 370
**9h**	Se	Cl	CH_3_	CH_3_	58% ± 7	5% ± 2	19% ± 5	1060 ± 140
**9i**	Se	Cl	3-F-Bn	CH_3_	34% ± 2	9% ± 4	14% ± 2	238 ± 37
**9j**	Se	Cl	3-Cl-Bn	CH_3_	63% ± 5	13% ± 1	27% ± 4	195 ± 40
**9k**	Se	Cl	3-Br-Bn	CH_3_	58% ± 2	10% ± 4	27% ± 6	180 ± 12
**9l**	Se	Cl	3-I-Bn	CH_3_	68% ± 2	10% ± 1	23% ± 3	253 ± 29

^a^ All binding experiments were performed using adherent mammalian cells stably transfected with cDNA encoding the appropriate hAR (A_1_AR and A_3_AR in CHO cells, A_2A_AR in Hela cells and A_2B_AR in HEK-293 cells). Binding was carried out using 2 nM [^3^H]DPCPX, 3 nM [^3^H]ZM241385, 25 nM [^3^H]DPCPX or 0.5 nM [^3^H]NECA as radioligands for A_1_, A_2A_, A_2B_, and A_3_ARs, respectively. Values are expressed as mean ± SEM (*n* = 2). ^b^ When a value is expressed as a percentage, it refers to the percent inhibition of a specific radioligand binding at 10 μM, with nonspecific binding defined using 10 μM NECA. ^c^ Ref [16]. ^d^ Ref [19].

## Data Availability

The data presented in this study are available in the article.
